# From Allergens to Battery Anodes: Nature-Inspired, Pollen Derived Carbon Architectures for Room- and Elevated- Temperature Li-ion Storage

**DOI:** 10.1038/srep20290

**Published:** 2016-02-05

**Authors:** Jialiang Tang, Vilas G. Pol

**Affiliations:** 1 School of Chemical Engineering, Purdue University, West Lafayette, IN 47907, USA

## Abstract

The conversion of allergic pollen grains into carbon microstructures was carried out through a facile, one-step, solid-state pyrolysis process in an inert atmosphere. The as-prepared carbonaceous particles were further air activated at 300 °C and then evaluated as lithium ion battery anodes at room (25 °C) and elevated (50 °C) temperatures. The distinct morphologies of bee pollens and cattail pollens are resembled on the final architecture of produced carbons. Scanning Electron Microscopy images shows that activated bee pollen carbon (ABP) is comprised of spiky, brain-like, and tiny spheres; while activated cattail pollen carbon (ACP) resembles deflated spheres. Structural analysis through X-ray diffraction and Raman spectroscopy confirmed their amorphous nature. X-ray photoelectron spectroscopy analysis of ABP and ACP confirmed that both samples contain high levels of oxygen and small amount of nitrogen contents. At C/10 rate, ACP electrode delivered high specific lithium storage reversible capacities (590 mAh/g at 50 °C and 382 mAh/g at 25 °C) and also exhibited excellent high rate capabilities. Through electrochemical impedance spectroscopy studies, improved performance of ACP is attributed to its lower charge transfer resistance than ABP. Current studies demonstrate that morphologically distinct renewable pollens could produce carbon architectures for anode applications in energy storage devices.

Since its first introduction by the Sony Corporation^1^ in 1991, lithium ion batteries (LIBs) remain as the dominating energy storage technology for portable electronics and electric vehicles. The traditional LIB anode is graphite, exhibiting a theoretical capacity of 372 mAh/g and excellent capacity retention over extended cycling. However, due to low operating voltage (<0.3 V vs. Li/Li^+^), graphite is subject to lithium plating and subsequently lithium dendrite formation when cycled at fast rates or low temperature^2^,^3^. To mitigate this potential safety hazard and to improve the rate capabilities of LIBs, significant research efforts have been devoted into identifying the next-generation anode materials for LIBs.

One such material is hard carbon which typically allows faster lithiation due to larger interlayer spacing, and higher cycling capacity than graphite due to the additional nanopore filling Li storage mechanism^4^. Experimentally, hard carbon of various morphologies such as spheres^5^, hollow spheres^6^,^7^, nanosheets^8^,^9^, carbon nanofibers^10^, and porous monolith^11^ have been prepared^12^,^13^. These carbon materials are commonly prepared by direct pyrolysis or hydrothermal decomposition of biomass^12^,^13^,^14^,^15^,^16^,^17^,^18^. *Wang et al*. prepared carbon fibers via hydrothermal carbonization of rice husk and used it as anode material for LIB and achieved capacity of ~400 mAh/g at 75 mA/g^19^. Moreover, *Lotfabad et al*. synthesized banana peel derived hard carbon via a pyrolysis reaction and reported high capacity of 1090 mAh/g in a LIB at current density of 50 mA/g^20^.

Aside from renewability, biomass sources can be carefully selected to fine tune their morphologies^12^,^15^,^21^. A vast biomass resource with wide selectivity of morphologies is pollen^22^. The significance of the varied pollen morphologies are seldom noticed by the general public due to their miniature sizes and their antagonistic role in allergy symptoms. Pollen grains typically have a tough outer layer made of sporopollenin biopolymer that is capable of deriving very divergent structures^22^. During the pollination season, plants can be thought to be minifactories that replicate and generate species-specific pollen grains. Due to the micrometer particle size of pollens (above 6 μm)^23^, considering their shrinkage after pyrolysis they could fall in the range of commerical carbon anode particle sizes. Solid, dense carbon particles provides high energy density to the rechargeable batteries.

Herein, we report the conversion of allergenic pollen grains into carbon microstructures through a facile, one-step, solid-state thermochemical decomposition in an inert atmosphere at elevated temperatures. For this study, carbon derived from both cattail pollens and bee pollens were evaluated for their potential application in LIB anodes. Cattail pollens was chosen to represent pollens with selective morphology but with limited commercial supply, while bee pollens represent pollens with unselective/diverse morphologies but are commercially available in large quantities.

## Results and Discussion

**Figure 1:**
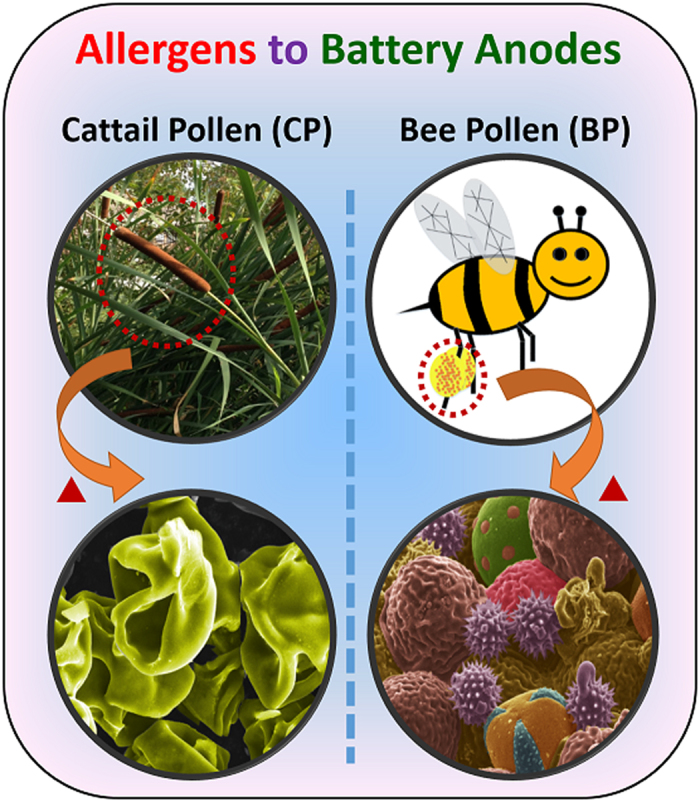
Preparation of pollen derived carbon microstructure via solid state pyrolysis of two distinct pollen sources.

**Figure 2:**
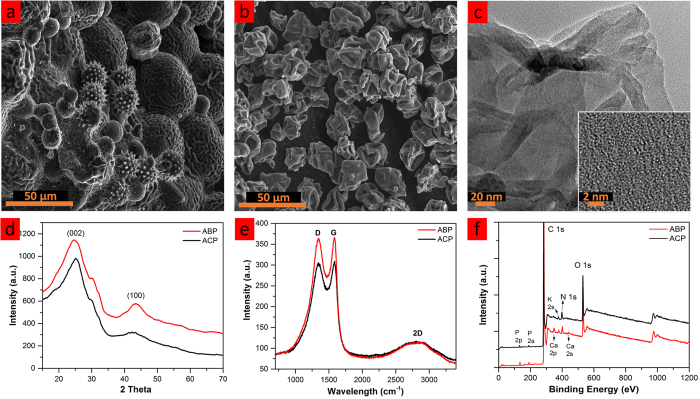
Characterization of pollen derived carbons. SEM images of (**a**) ABP and (**b**) ACP; (**c**) TEM images of ACP; (**d**) XRD and (**e**) Raman patterns of ABP and ACP; (**f**) XPS spectra of ground ABP and ACP samples.

Figure 1 illustrates the two pollen sources utilized in this study and their correponding carbon architecture. The cattail pollens were obtained from locally grown cattail plants, while the bee pollens were initally collected from flowers by foraging bees. The colored scanning electron microscopy (SEM) images of ACP and ABP reveal the distinct morphological difference of the two pollen derived carbons. ACP carbon resembles the shape of collapsed spheres with uniform diameter of ~20 μm (Fig. 2b). The uniformity is due to the utilization of a single pollen source. On the other hand, ABP exhibits unique morphologies of different pollen grains such as spiky spheres (~15 μm in diameter), brain-like spheres (~50 μm), and small smooth spheres (~10 μm) (Fig. 2a). The rich morphologies of ABP carbon can be attributed to the diverse pollen sources visited by the foraging bees. It is important to note that change of seasons and sourcing locations are expected to significantly impact the composition of bee pollens due to the change in available pollen species^24^. Such variation in bee pollen derived carbon microstrutures could pose considerable challenges to battery anode quality control; however, controllable carbon mictrostructures with monodispersity could be attained via careful selection of pollen sources. For scale-up production of pollen carbon, it is possible to obtain structural uniformity through utlization of bee pollens harvested from commerical pollination beehives with singular target crop.

Transmission electron microscopy (TEM) images were taken to reveal high degree of disorderness in ACP carbon as shown in Fig. 2c. Due to the diverse carbon microstrutures presented in ABP sample, TEM images of small sampling points would not be representative of the entire ABP sample hence its TEM were not collected.

Instead, X-ray diffraction and Raman spectroscopy were utlized to charaterize the bulk samples (Fig. 2d,e). XRD spectra reveal that ABP and ACP have very similar crystal structures with (002) peaks at 24.6° and 25.1° respectively, corresponding to interlayer spacing of 0.358 nm and 0.360 nm (vs. 0.335 nm for graphite^20^). The larger interlayer spacing is a common feature of hard carbon and is expected for these pollen samples since they were both synthesized at low temperatures. Moreover, Raman spectra of ABP and ACP both shows I_G_/I_D_ ratios of ~1.00, indicating the equivalent presence of both disordered and graphitic carbon in the samples. The similarities of XRD and Raman spectra between these two samples suggest analogous carbon composition and carbon crystal structure in both samples.

X-ray photoelectron spectroscopy (XPS) was carried out to further examine their compositional difference and the results are presented in Fig. 2f and Table 1. Since XPS is surface sensitive technique, prior to XPS measurements; both ABP and ACP samples were mechanically ground to expose their core composition^20^. Both ABP and ACP samples contain comparable amount of C contents (~83.5%) and slight deviations in O (~11.5%) and N (~3.0%) contents as listed in Table 1. The high level of oxygen presence in both samples likely resulted from air activation. It is known that air activiation in hard carbon improves lithiation capacity via formation of additional surface pores for additional lithiation^25^. Similar improvements in capacity have also been observed in current work as described in the following section. Additionally, trace amount of phosphorus (~1%), calcium (~0.5%), and potassium (~0.5%) are detected in both samples, as pollens naturally contain these components.

**Figure 3:**
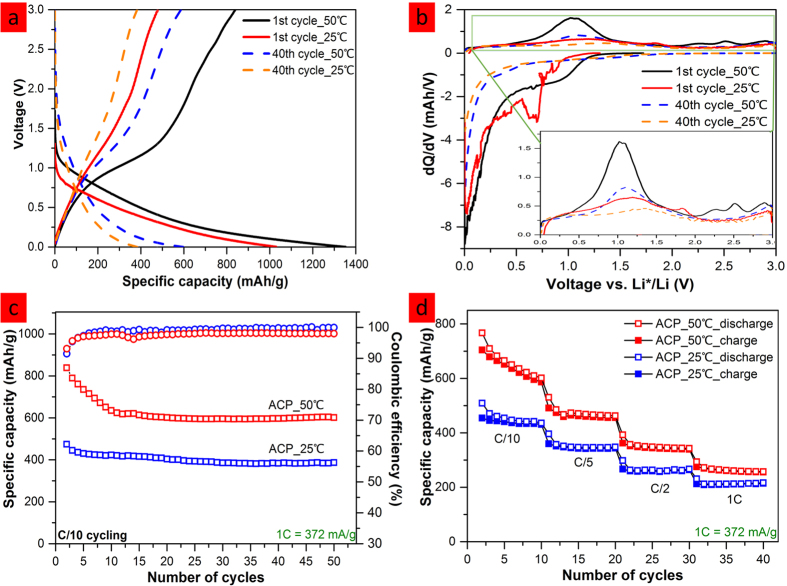
Electrochemical analysis of ACP vs Li half-cells. (**a**) Voltage profiles from 0 to 3 V at C/10 rate for both 50 °C and 25 °C cells; (**b**) corresponding dQ/dV plots, inset is the enlarged charge plots; (**c**) capacity as a function of cycle number at C/10 rate; (**d**) rate studies of ACP vs Li at room and elevated temperatures.

Long cycling of electrode comprising ACP vs. Li half cells were studied at C/10 rate within voltage window of 0 to 3 V at both 50 °C and 25 °C. Voltage profiles of both cells were constructed to compare their cycling behavior (Fig. 3a). Both cells show sloping voltage profiles that are characteristic of hard carbon^5^; low-voltage plateaus that is typically seen in graphite anode is not observed here, suggesting the risk of lithium plating could be reduced. Furthermore, cattail pollen-derived anode cycled at elevated temperature (ACP_50 °C) exhibit slightly higher potential onset at 1^st^ and 40^th^ discharges than a similar anode cycled at room temperature (ACP_25 °C), suggesting a greater tendency for ACP to lithiate at elevated temperature.

Differential capacity (dQ/dV) plots of the 1^st^ and 40^th^ cycles for each cell further reveal the difference in their cycling behavior. As shown in Fig. 3b, the 1^st^ discharge curve of ACP_25 °C cell appears to be erratic from 1.0V to 0.2V; this can be correlated to the 1^st^ cycle solid electrolyte interphase (SEI) formation^26^. On the contrary, ACP_50 °C cell exhibits rather smooth discharge curve, suggesting very different reaction pathways to form the initial SEI layer, likely promoted by the temperature elevation. The continuous capacity fading observed in the first 10 cycles of ACP_50 °C cell suggests that this 1^st^ cycle SEI layer is not stable (Fig. 3c). The 40^th^ discharge curves for both cells represent stable discharge profiles at both 50 °C and 25 °C; and their comparable profiles suggest similar lithium intercalation mechanisms into the ACP carbon.

On the other hand, 1^st^ and 40^th^ charge profiles of both cells are shown on the inset of Fig. 3b to reveal distinctly different deintercalation features at the two temperatures. The peaks centered at 1–1.3 V region are narrower and more intense for the 50 °C cell than the 25 °C cell, indicating a more moderate sloping profile at higher temperature^27^ as observed in Fig. 3a. Small peaks appears at 2–3 V in the 1^st^ charge curve of the 50 °C cell may be attributed to the extraction of Li from oxygen functional groups that reside on the surface of carbonaceous materials^28^,^29^,^30^. These peaks disappear upon cycling likely due to the deactivation of surface functional groups.

Figure 3c shows the long cycling performance of both ACP_50 °C and ACP_25 °C cells at C/10 rate. ACP_50 °C cell exhibits high capacity of 590 mAh/g with minimal capacity fading after the first 10 cycles, while ACP_25 °C cell reaches stable reversible capacity of 382 mAh/g after the 1^st^ cycle. The higher capacity of ACP_50 °C cell can be attributed to faster charge transfer/Li ion diffusion at elevated temperature enabling better utilization of the active material. The longer time it took to reach stable capacity is a result of more side reactions taking place on the carbon surface at elevated temperature until a thicker and more stable SEI layer is formed^31^. This phenomena is also reflected on the lower coulombic efficiency of ACP_50 °C cell (~97%).

Further studies were conducted to evaluate ACP cells’ rate capability at 50 °C and 25 °C. As shown in Fig. 3d, ACP_50 °C cell displays significant improvements in capacities (up to 200 mAh/g more capacity) at slow cycling rates, likely due to the fast charge transfer/Li ion diffusion at elevated temperature. However, the advantage of high temperature gradually diminishes at faster cycling rates. In terms of cycling stability, 25 °C cell outperforms 50 °C cell at slow cycling rates as a result of having fewer side reactions at lower temperature^26^,^31^.

**Figure 4:**
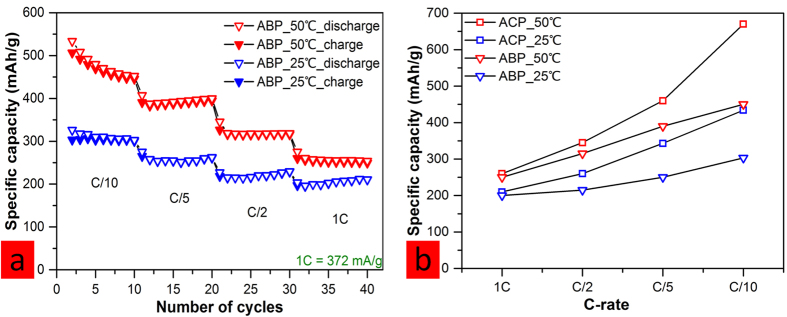
(**a**) Rate studies of ABP half-cells from 0 to 3 V at both 50 °C and 25 °C; (**b**) comparison of all rate studies of ACP and ABP cells.

ABP half-cells were also cycled to evaluate their performance against ACP cells. As shown in Fig. 4a, the rate study of ABP cells at both 50 °C and 25 °C reveal encouraging results considering the fact that these active material morphologies were not optimized or controlled. When compared to the ACP cells data (Fig. 4b), ABP cells, as expected, exhibit lower capacities (~150 mAh/g lower at C/10 rate for both high and low temperatures) at all rates and at each temperature. The difference in electrochemical performance could be a result of morphological difference between ABP and ACP carbon. As revealed in SEM images, ACP contains particles with smooth surface while ABP carbon contains spheres with various morphologies including ones with spikes which could have led to high local current density that exacerbates side reactions and excessive SEI buildup^32^. Moreover, this morphological difference likely gives rise to the larger BET surface area of ABP carbon (303.9 m^2^/g) in comparison to ACP carbon (237.6 m^2^/g) as listed in Table 2 below (also see Supplementary Fig. S2). The larger surface area could translate to larger electrolyte-electrode reaction interface, which had been linked to more SEI formation^26^. Consequently, ABP cells show higher 1^st^ irreversible capacity losses at both 50 °C and 25 °C.

**Figure 5:**
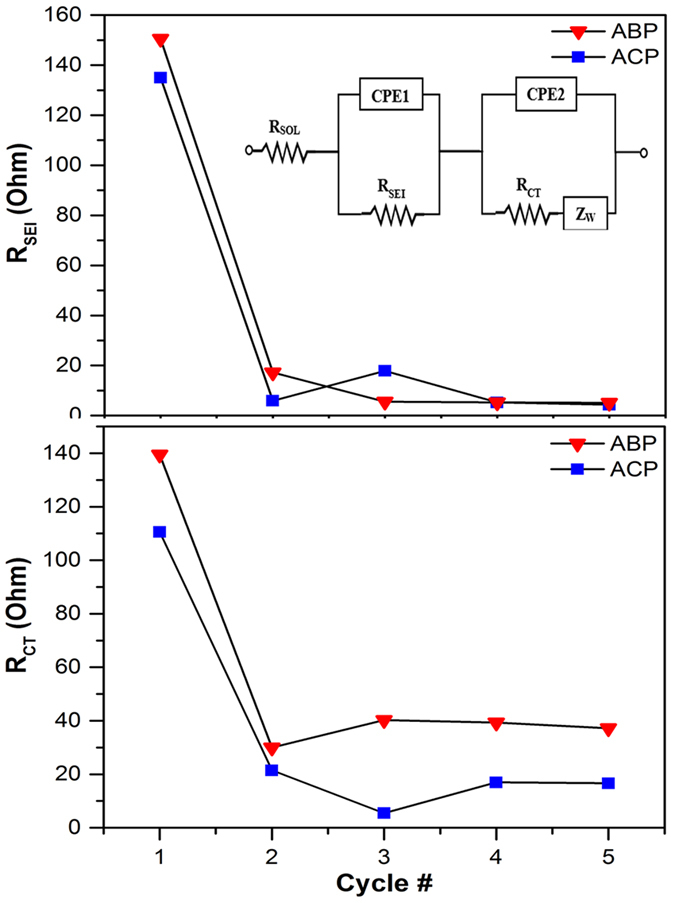
SEI and charge transfer resistances at 0.5 V discharges as a function of cycles. The inset shows the equivalent circuit used to model the EIS data.

Electrochemical impedance spectrpscopy (EIS) was employeed to probe the evolution of SEI and charge transfer kinetics of both pollen carbons at room temperature with respect to time. The inset of Fig. 5 illustrates the equivalent circuit model used to fit the EIS data. R_SOL_ is the resistance of the electrolyte solution and R_SEI_ is the SEI film resistance. The constant phase element (CPE) was used instead of double-layer capacitor to account for surface nonideality (roughness) of the particle^33^. R_CT_ is the charge transfer resistance at the particle surface. And Z_W_ is the Warburg impedance that describes solid state diffusion of lithium ions into carbon. All the EIS and fitting data are summerized in Fig. S3. R_SOL_ calculated from the model remain nearly constant (~2.6 Ω) for all the measurements. The results for R_SEI_ and R_CT_ are summerized in Fig. 5. Both ABP and ACP cells experienced high film resistance (R_SEI _= 150 Ω and 135 Ω, respectively) during the 1^st^ lithiaion as a result of initial SEI formation. The slightly greater value of ABP cell may come from its larger surface area as discussed previously. The film resistance for both cells dropped significantly after the initial discharge and quickly stablized to ~5 Ω after 3 cycles, suggesting the formation of stable SEI layers on both pollen derived carbon. The charge transfer resistance R_CT_ exhibited very similar behaviors for both cells during the first two cycles and stablized at ~39 Ω for ABP and ~16 Ω for ACP afterward. In other words, ABP pacticles experiences more than twice the charge transfer resistance of ACP. Since the film resistances are very similar in both cells, difference in SEI is unlikely the root cause for difference in their charge transfer resistance^34^. One possible explanation is that ABP derived carbon is low in N contents, which have been known to improve surface reactivity and electron conductivity in carbon^35^,^36^. Consequently, ABP should have a lower electron conductivity leading to poorer charge transfer than ACP. However, the opposite observation may also be true if high P content (also benefits charge transfer^37^) in ABP is taken into account. Nevertheless, the difference in R_CT_ is in good agreement with the galvanostatic cycling results (Fig. 4).

In summary, carbon microstrutures with unique morphologies have been successfully sythesized from bee pollens and cattail pollens through a facile, one-step solid-state pyrolysis route. XRD and Raman spectroscopy were employeed to confirm their hard carbon nature; and XPS elemental analysis revealed that both samples contain high levels of O content (~11.5%), which contributes to their excellent capacities and rate capabilities. ACP carbon were found to deliver high capacities of 590 mAh/g at 50 °C and 382 mAh/g at 25 °C when cycled at C/10 rate, they also exhibit excellent rate capabilities with 1C rate. ABP cells were cycled under the same conditions but were found to deliver less capacities than ACP cells. The capacity difference between ABP and ACP are up to 150 mAh/g for both temperatures. EIS model fitting shows that ACP carbon benefits from having lower charge transfer resistance which is likely influenced by the presence of conductivity-enhancing elements. This study explored the potential of utilizing pollens as renewable carbon sources for energy storage application and found that high reversible capacities are achievable with careful selection of pollen morphology.

## Methods

### Synthesis of carbon microstructure

Bee pollen granules (Stakich, 1 pound) were used as purchased; cattail pollens were collected from blooming cattail spikes grown locally on August 2014. Each pollen sample was dispersed in deionized water and filtered through a 0.5 mm sieve to remove non-pollen impurities; this procedure was repeated three times. After drying overnight at 80 °C in a vacuum oven, these samples were pyrolyzed at 600 °C for 3 hours in a tube furnace under continuous argon gas flow (based on the TGA results Fig. S1). The obtained samples were subsequently activated in air at 300 °C for 6 hours in a tube furnace. The activated bee-pollen carbon is denoted as “ABP” while activated cattail pollen is denoted as “ACP”.

### Electrochemical Testing

Electrode slurry consisting of 80 wt% ABP or ACP, 10 wt% carbon black, and 10 wt% polyvinylidene fluoride (PVDF) dissolved in appropriate amount of n-methly-2-pyrrolidone (NMP) solvent was homogenized for 30 mins using a Thinky mixer. The slurry was subsequently casted onto a copper film using a doctor-blade. The laminate was dried overnight at 80 °C in a vacuum oven and then punched into 12 mm diameter electrodes. The as-prepared electrode was paired with a lithium counter electrode separated by a polymer membrane (Celgard 2500) in CR2032 coin cells. The electrolyte used is 1M LiPF_6_ in equal volumetric ratio of ethylene carbonate (EC), diethyl carbonate (DEC), and dimethyl carbonate (DMC). Galvanostatic cycling was conducted using Arbin BT-2000 Potentiostat at both 25 °C and 50 °C (inside ESPEC BTZ environmental chamber), henceforth described as room temperature and elevated temperature, respectively. EIS measurements were carried out on a Gamry Reference 600 Electrochemical Workstation. Both cells were cycled at C/2 rate (with 1C being 372 mAh/g) in room temperature. The impedance data were obtained at 0.5 V during each discharge by applying AC voltage perturbation with amplitude of 10 mV over frequency range from 1MHz to 0.01 HZ. Fitting of EIS results was performed using Gamry Echem Analyst software and goodness of fit for all fitting is in the order of 10^−3^.

### Material Characterization

*FEI Nova 200 NanoLab DualBeam TM-SEM/FIB* was utilized to obtain Scanning Electron Microscopy (SEM) images of the samples. *FEI-TITAN* microscope operating at an accelerating voltage of 300 kV was used to obtain Transmission Electron Microscopy (TEM) images. X-ray diffraction was collected from 10° to 80° at 5 degree/min scan rate using *Rigaku SmartLab XRD* with a Cu Kα radiation source. *Thermo Scientific DXR Raman Microscope* with a 532-nm laser was used for Raman measurements. Kratos X-ray photoelectron spectrometer was employed for XPS measurement. Prior to XPS analysis, the samples were mechanically ground and dried at 80 °C overnight under vacuum. The process of thermal decomposition of pollens were evaluated from 30 °C to 800 °C at 10 °C/min heating rate under constant helium flow (100 ml/s) using a *TA DST Q600* thermal gravimetric analyzer. Nitrogen sorption measurement at 77K was conducted using Quantachrome Nova 2200e surface analyzer. Prior to analysis, the samples were degassed at 300 °C for 12 hours.

## Additional Information

**How to cite this article**: Tang, J. and Pol, V. G. From Allergens to Battery Anodes: Nature-Inspired, Pollen Derived Carbon Architectures for Room- and Elevated- Temperature Li-ion Storage. *Sci. Rep*. **6**, 20290; doi: 10.1038/srep20290 (2016).

## Supplementary Material

Supplementary Information

## Figures and Tables

**Table 1:** XPS elemental composition comparison of pollen carbons.

Samples	C	O	N	P	Ca	K
	*wt%*	*wt%*	*wt%*	*wt%*	*wt%*	*wt%*
*ABP*	84.08	10.81	2.74	1.31	0.73	0.44
*ACP*	83.18	12.14	3.20	0.82	0.12	0.55

**Table 2:** Surface characterization of pollen carbons vs. 1^st^ cycle irreversible capacity loss.

Samples	BET Surface Area (m^2^/g)	DFT Pore Diameter (Mode) (nm)	1st cycle irreversible (mAh/g)	
			Room Temp.	High Temp.
*ABP*	303.9	1.543	614.9	590.2
*ACP*	237.6	1.475	556.7	582.9

## References

[b1] Yang Z. . Electrochemical energy storage for green grid. Chem. Rev. 111, 3577–3613 (2011). 21375330 10.1021/cr100290v

[b2] Downie L. E. . *In Situ* Detection of Lithium Plating on Graphite Electrodes by Electrochemical Calorimetry. J. Electrochem. Soc. 160, 588–594 (2013).

[b3] Li Z., Huang J., Yann Liaw B., Metzler V. & Zhang J. A review of lithium deposition in lithium-ion and lithium metal secondary batteries. J. Power Sources 254, 168–182 (2014).

[b4] Stevens D. A. & Dahn J. R. The Mechanisms of Lithium and Sodium Insertion in Carbon Materials. J. Electrochem. Soc. 148, A803 (2001).

[b5] Pol V. G. & Thackeray M. M. Spherical carbon particles and carbon nanotubes prepared by autogenic reactions: Evaluation as anodes in lithium electrochemical cells. Energy Environ. Sci. 4, 1904 (2011).

[b6] Deshmukh A. a., Mhlanga S. D. & Coville N. J. Carbon spheres. Mater. Sci. Eng. R 70, 1–28 (2010).

[b7] Tang K. . Hollow Carbon Nanospheres with Superior Rate Capability for Sodium-Based Batteries. Adv. Energy Mater. 2, 873–877 (2012).

[b8] Etacheri V., Hong C. N. & Pol V. Upcycling of Packing-Peanuts into Carbon Microsheet Anodes for Lithium-Ion Batteries. Environ. Sci. Technol. 150622140953009 doi: 10.1021/acs.est.5b01896 (2015). 26098219

[b9] Wang H. . Interconnected Carbon Nanosheets Derived from Hemp for Ultrafast Supercapacitors with High Energy. ACS Nano 7, 5131–5141 (2013). 23651213 10.1021/nn400731g

[b10] Luo W. . Carbon nanofibers derived from cellulose nanofibers as a long-life anode material for rechargeable sodium-ion batteries. J. Mater. Chem. A 1, 10662–10666 (2013).

[b11] Adelhelm P., Cabrera K. & Smarsly B. M. On the use of mesophase pitch for the preparation of hierarchical porous carbon monoliths by nanocasting. Sci. Technol. Adv. Mater. 13, 15010 (2012).

[b12] Hu B. . Engineering carbon materials from the hydrothermal carbonization process of biomass. Adv. Mater. 22, 813–828 (2010). 20217791 10.1002/adma.200902812

[b13] Titirici M.-M. . Sustainable carbon materials. Chem. Soc. Rev. 44, 250–290 (2015). 25301517 10.1039/c4cs00232f

[b14] Titirici M. M. . A Direct Synthesis of Mesoporous Carbons with Bicontinuous Pore Morphology from Crude Plant Material by Hydrothermal Carbonization. Chem. Mater. 19, 4205–4212 (2007).

[b15] Hu B., Yu S.-H., Wang K., Liu L. & Xu X.-W. Functional carbonaceous materials from hydrothermal carbonization of biomass: an effective chemical process. Dalt. Trans. 9226, 5414–5423 (2008). 10.1039/b804644c 19082021

[b16] Yanik J., Stahl R., Troeger N. & Sinag A. Pyrolysis of algal biomass. J. Anal. Appl. Pyrolysis 103, 134–141 (2013).

[b17] Byrappa K. & Adschiri T. Hydrothermal technology for nanotechnology. Prog. Cryst. Growth Charact. Mater. 53, 117–166 (2007).

[b18] Xia Y. . Biotemplated fabrication of hierarchically porous NiO/C composite from lotus pollen grains for lithium-ion batteries. J. Mater. Chem. 22, 9209 (2012).

[b19] Wang L., Schnepp Z. & Titirici M. M. Rice husk-derived carbon anodes for lithium ion batteries. J. Mater. Chem. A 1, 5269–5273 (2013).

[b20] Lotfabad E. M. . High-Density Sodium and Lithium Ion Battery Anodes from Banana Peels. ACS Nano 8, 7115–7129 (2014). 24897543 10.1021/nn502045y

[b21] Falco C., Baccile N. & Titirici M.-M. Morphological and structural differences between glucose, cellulose and lignocellulosic biomass derived hydrothermal carbons. Green Chem. 13, 3273 (2011).

[b22] McCormick S. Pollen. Curr. Biol. 23, 988–990 (2013). 10.1016/j.cub.2013.08.016 24262831

[b23] Knight C. a., Clancy R. B., Götzenberger L., Dann L. & Beaulieu J. M. On the Relationship between Pollen Size and Genome Size. J. Bot. 2010, 1–7 (2010).

[b24] Yang K. . Characterization of chemical composition of bee pollen in China. J. Agric. Food Chem. 61, 708–718 (2013). 23265625 10.1021/jf304056b

[b25] Fujimoto H., Tokumitsu K., Mabuchi A., Chinnasamy N. & Kasuh T. The anode performance of the hard carbon for the lithium ion battery derived from the oxygen-containing aromatic precursors. J. Power Sources 195, 7452–7456 (2010).

[b26] Xu K. Nonaqueous Liquid Electrolytes for Lithium-Based Rechargeable Batteries. Chem. Rev. 104, 4303–4418 (2004). 15669157 10.1021/cr030203g

[b27] Wu G. T. . Structure and lithium insertion properties of carbon nanotubes. J. Electrochem. Soc. 146, 1696–1701 (1999).

[b28] Klink S. . Tailoring of CNT surface oxygen groups by gas-phase oxidation and its implications for lithium ion batteries. Electrochem. commun. 15, 10–13 (2012).

[b29] Ventosa E. . Influence of surface functional groups on lithium ion intercalation of carbon cloth. Electrochim. Acta 65, 22–29 (2012).

[b30] Oktaviano H. S., Yamada K. & Waki K. Nano-drilled multiwalled carbon nanotubes: characterizations and application for LIB anode materials. J. Mater. Chem. 22, 25167 (2012).

[b31] Leng F., Tan C. M. & Pecht M. Effect of Temperature on the Aging rate of Li Ion Battery Operating above Room Temperature. Sci. Rep. 5, 12967 (2015). 26245922 10.1038/srep12967 PMC4526891

[b32] Pol V. G. . Spherical Carbon as a New High-Rate Anode for Sodium-ion Batteries. Electrochim. Acta 127, 61–67 (2014).

[b33] Umeda M. . Electrochemical impedance study of Li-ion insertion into mesocarbon microbead single particle electrode Part II. Disordered carbon. Electrochim. Acta 47, 885–890 (2001).

[b34] Yang S., Huo J., Song H. & Chen X. A comparative study of electrochemical properties of two kinds of carbon nanotubes as anode materials for lithium ion batteries. Electrochim. Acta 53, 2238–2244 (2008).

[b35] Qie L. . Nitrogen-Doped Porous Carbon Nanofiber Webs as Anodes for Lithium Ion Batteries with a Superhigh Capacity and Rate Capability. Adv. Mater. 24, 2047–2050 (2012). 22422374 10.1002/adma.201104634

[b36] Wu Z.-S., Ren W., Xu L., Li F. & Cheng H.-M. Doped Graphene Sheets As Anode Materials with Superhigh Rate and Large Capacity for Lithium Ion Batteries. ACS Nano 5, 5463–5471 (2011). 21696205 10.1021/nn2006249

[b37] Zhang C., Mahmood N., Yin H., Liu F. & Hou Y. Synthesis of Phosphorus-Doped Graphene and its Multifunctional Applications for Oxygen Reduction Reaction and Lithium Ion Batteries. Adv. Mater. 25, 4932–4937 (2013). 23864555 10.1002/adma.201301870

