# Corrigendum: From Allergens to Battery Anodes: Nature-Inspired, Pollen Derived Carbon Architectures for Room- and Elevated- Temperature Li-ion Storage

**DOI:** 10.1038/srep32276

**Published:** 2016-09-02

**Authors:** Jialiang Tang, Vinodkumar Etacheri, Vilas G. Pol

Scientific Reports 6: Article number: 2029010.1038/srep20290; published online: 02 05 2016; updated: 09 02 2016

Vinodkumar Etacheri was omitted from the author list in the original version of this Article. This has now been corrected in the PDF and HTML versions of the Article, as well as the Supplementary Information file that now accompanies the Article.

The Acknowledgements section now reads:

The authors thank Purdue University and its School of Chemical Engineering for their generous start-up funding. Electron microscopy studies at Birck nanotechnology center were funded by Kirk exploratory research grant and were conducted by Arthur D. Dysart (SEM). XPS measurement was conducted by Dr. Dmitry Zemlyanov at Brick nanotechnology center. We also thank Thermo Scientific for DXR Raman microscope. J. Tang is indebted to Hoosier Heavy Hybrid Center of Excellence (H3CoE) fellowship (funded by US Department of Energy) for its financial support.

The Author Contributions section now reads:

J.T. conceived and conducted the experiment; V.E. collected the TEM images; J.T. and V.G.P. analyzed the data; J.T. wrote the manuscript and V.G.P. revised the manuscript.

