# Supplementary material for: From Allergens to Battery Anodes: Nature-Inspired, Pollen Derived Carbon Architectures for Room- and Elevated- Temperature Li-ion Storage
Source: Sci Rep. 2016 Feb 5;6:20290. doi: 10.1038/srep20290 (PMC4742870; doi:10.1038/srep20290)
Supplement: Supplementary Information [file srep20290-s1.pdf]

From Allergens to Battery Anodes: Nature-Inspired, Pollen Derived Carbon Architectures for Room- and Elevated- Temperature Li-Ion Storage

Jialiang Tang[1]
Tang186@purdue.edu
(512) 731-9093

Vilas G. Pol[1]*
Vpol@purdue.edu
(765) 494-0044

1. School of Chemical Engineering, Purdue University, West Lafayette, IN 47907, USA.

Thermal gravimetric analysis (TGA) was conducted to study the carbonization process of the pollen samples. It was found that bee pollens and cattail pollens lost about 75% and 80% of their original mass respectively upon carbonization. The primary pyrolysis step occurs in the region between ~200 ℃ and ~500 ℃, where the breakdown of protein, lipids, fibers, and other organic compounds take place[1]. At around 600°C, most of hydrocarbons have been converted to carbonaceous moieties and further weight loss is minimal; therefore, ACP and ABP thermal processing were conducted at 600°C.

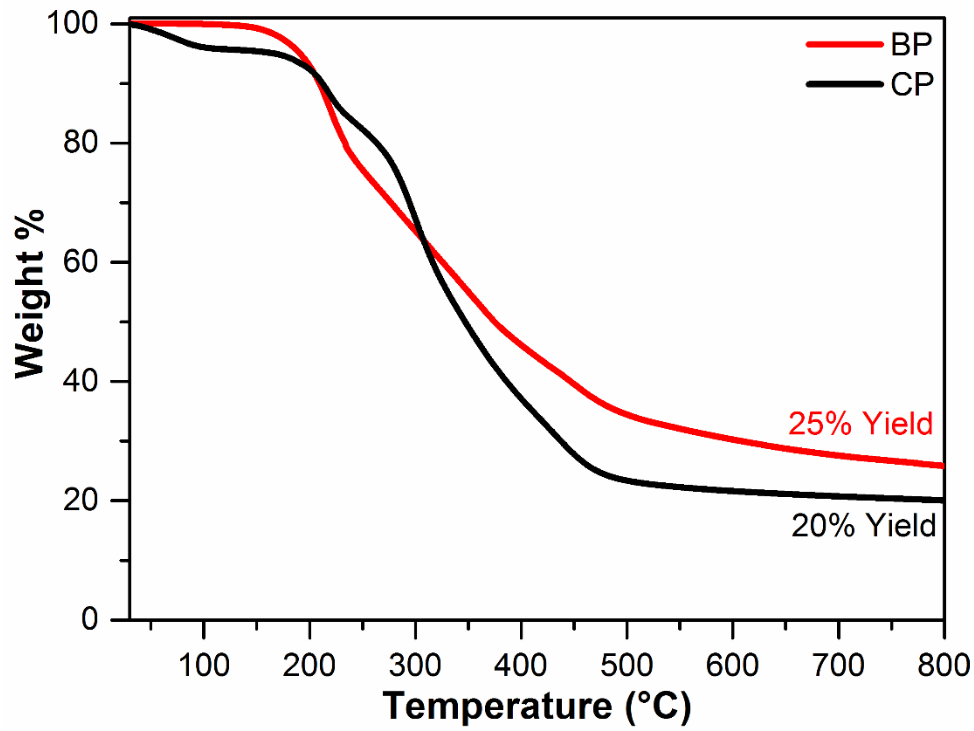

Supplementary Figure S1: TGA curves of bee pollen and cattail pollen.

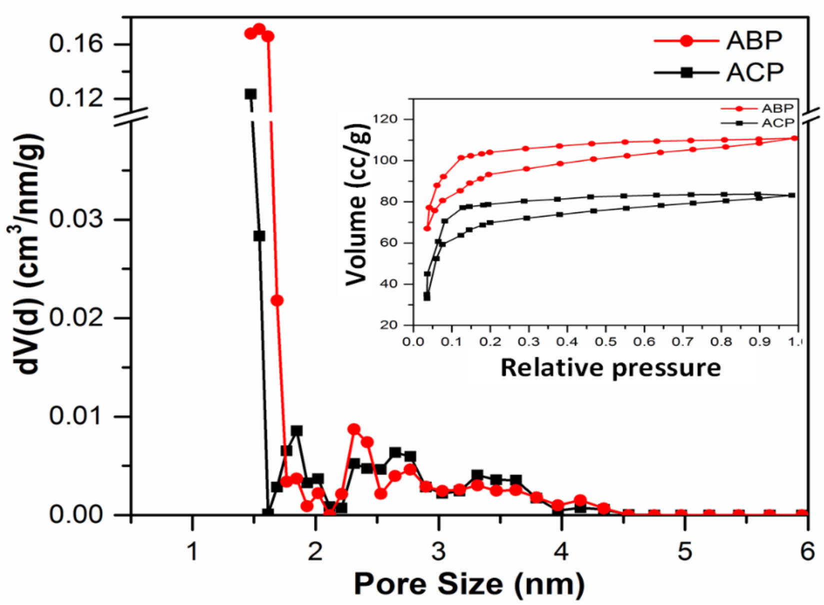

Supplementary Figure S2: Nitrogen adsorption measurements at 77K. Pore size distribution was calculated from adsorption isotherms using the DFT method. The inset shows the adsorption and desorption isotherms of ABP and ACP.

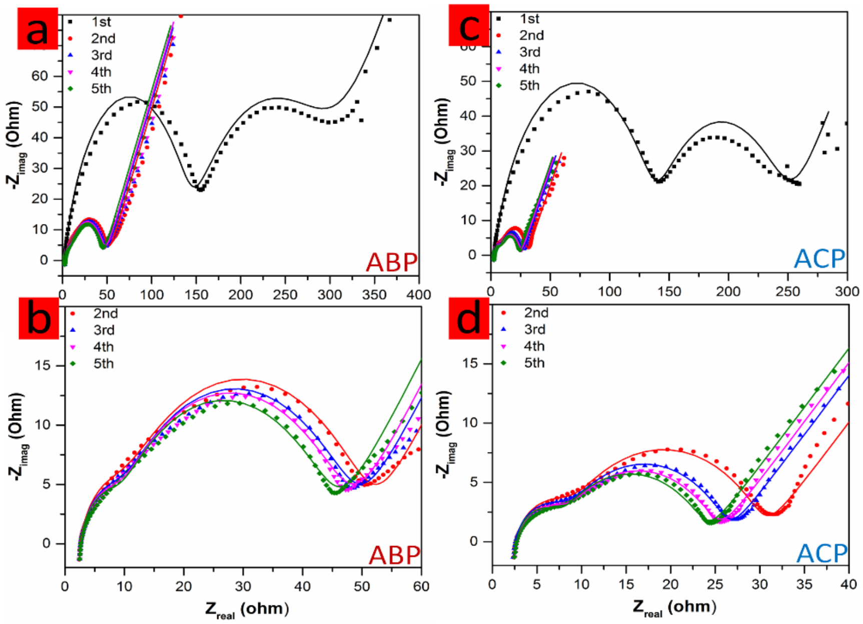

Supplementary Figure S3: all EIS spectra (symbols) at 0.5V and corresponding fitting (solid lines). a) & b) ABP; c) & d) ACP.

Reference

(1) Campos, M. G. R.; Bogdanov, S.; de Almeida-Muradian, L. B.; Szczesna, T.; Mancebo, Y.; Frigerio, C.; Ferreira, F. Pollen composition and standardisation of analytical methods. *J. Apic. Res.* **2008**, *47* (2), 154–161.
